# Perspective: Vibronic Coupling Potentials for Trajectory-Based Excited-State Dynamics

**DOI:** 10.1021/acs.jctc.5c01002

**Published:** 2025-09-11

**Authors:** Sandra Gómez, Patricia Vindel-Zandbergen, Dilara Farkhutdinova, Leticia González

**Affiliations:** † Departamento de Química, Módulo 13, Universidad Autónoma de Madrid, Cantoblanco, Madrid 28049, Spain; ‡ Department of Chemistry, New York University, New York, New York 10003, United States; § Simons Center for Computational Physical Chemistry at New York University, New York, New York 10003, United States; ∥ Institute of Theoretical Chemistry, Faculty of Chemistry, 27258 University of Vienna, Währinger Str. 17, Vienna 1090, Austria; ⊥ Vienna Doctoral School in Chemistry (DoSChem), 27258 University of Vienna, Währinger Straße 42, Vienna 1090, Austria; # Vienna Research Platform on Accelerating Photoreaction Discovery, 27258 University of Vienna, Währinger Straße 17, Vienna 1090, Austria

## Abstract

This Perspective reviews the use of vibronic coupling (VC) potentials in trajectory-based excited-state dynamics simulations. Originally developed to provide simplified yet physically grounded representations of nonadiabatic interactions, VC models—particularly their linear version (LVC)—have facilitated extensive investigations of photophysical and photochemical processes, in both molecular and condensed-phase systems. By effectively capturing the coupling between electronic and vibrational motions, VC models enable efficient dynamical simulations, making it feasible to investigate larger and more complex systems, for longer time scales or relying on potential energy surfaces calculated with high levels of theory. These models provide valuable insights into energy and charge transfer mechanisms following photoexcitation, shedding light on excited-state lifetimes and intricate relaxation pathways. Here, we discuss their integration with three trajectory-based computational families of methods: surface hopping, variational multiconfigurational Gaussian, and exact-factorization-derived approaches. We showcase how VC models have helped uncovering key mechanistic insights, including state-specific intersystem crossing pathways and vibrational mode selectivity. As the field progresses, VC-based approaches are expected to be increasingly combined with machine learning, anharmonic corrections, and hybrid LVC/MM frameworks, broadening their applicability to complex, flexible, and solvated environments. We highlight the advantages of VC-based potentials for trajectory-based simulations, emphasizing their computational efficiency and usefulness for benchmarking and exploring photophysical processes in molecular systems.

## Graphical abstract

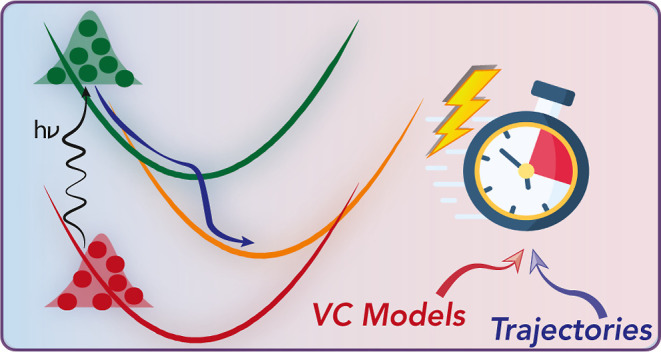

## 1 Introduction

Excited-state dynamics explores the ultrafast motion of nuclei and electrons in molecules following the absorption of light. Understanding the fate of this coupled nuclear-electron behavior is essential for interpreting a wide range of photochemical reactions, in systems that range from organic molecules to biological complexes and materials.^1,2^ An attractive computational strategy to investigate such processes is the use of direct molecular dynamics methods, also known as on-the-fly molecular dynamics, where potential energy surfaces (PESs) are evaluated in real time as the nuclei evolve. In trajectory-based approaches,^3–5^ at each time step, an external electronic structure program is typically called to compute the energies and forces acting on the nuclei, as well as nonadiabatic couplings, and when required, spin–orbit couplings or other relevant quantities (Figure 1a). The PES underlying excited-state dynamics simulations can be obtained using various methods,^6,7^ including semiempirical approaches,^8^ time-dependent density functional theory (TD-DFT),^9,10^ or ab initio methods, among which multireference configuration interaction singles and doubles (MR-CISD),^11^ complete active space self-consistent field (CASSCF),^12^ algebraic diagrammatic construction (ADC) methods,^13^ and complete active space perturbation theory (CASPT2),^14^ are probably the most employed. The popularity of direct dynamics stems from its flexibility and accuracy in handling complex dynamical changes driven by nuclear motion in molecules. Direct dynamics is particularly valuable for investigating flexible molecules that undergo large conformational transformations, such as photoisomerization, as well as photochemical reactions that involve bond breaking and formation,^15,16^ thus providing valuable insight into the intricate pathways and/or energy barriers that govern light-induced processes.

**Figure 1:**
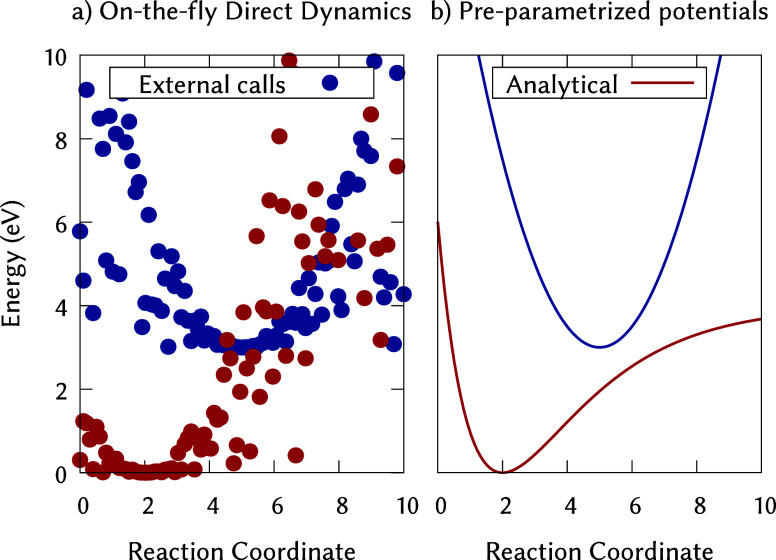
Comparison of two approaches to excited-state molecular dynamics simulations. (a) On-the-fly (direct) dynamics: potential energies are computed at each geometry by an external electronic structure package, yielding discrete energy points (red and blue) along a reaction coordinate in full dimensionality. (b) Parametrized potentials: potential energy surfaces are precomputed and described by analytical expressions (red and blue curves) on which dynamics is subsequently performed. Note that if the direct dynamical results would be projected onto a selected coordinate, the energies would align with the analytical curves.

In contrast to direct dynamics, excited-state simulations based on preparameterized potentials rely on fitting global representations of the PES at selected geometries before the propagation of the dynamics (Figure 1b). Once constructed, these surfaces enable rapid trajectory propagation because the PES remains fixed during the simulation. This approach is computationally less demanding than direct dynamics, allowing simulations over long time scales, the propagation of large ensembles of trajectories and the use of higher level in the electronic structure methods during the PES construction. However, the precomputation of the full PES involves a substantial upfront cost and is typically feasible only for systems with a limited number of degrees of freedom or well-characterized reactive pathways that allow to simplify a full-dimensional PES to few degrees of freedom. To overcome these limitations, several strategies have been developed to construct simplified yet physically motivated PES, offering an efficient compromise that enables the study of large systems or long-time dynamics with reduced computational demands, where on-the-fly calculations would otherwise be prohibitive.

Vibronic coupling (VC) models^17–19^ are one of such theoretical frameworks that can be used to approximate the molecular Hamiltonian describing the interaction between electronic and vibrational degrees of freedom. In these models, the electronic states of a molecule are coupled to its vibrational modes, allowing for the study of electronic transitions along the associated changes in molecular geometry and vibrational motion, providing a simple representation of the PES. In the simplest of such models, the coupling between the electronic states and the vibrational modes is considered linear and thus termed linear VC (LVC),^20^ meaning that the strength of the coupling is proportional to the displacement of the vibrational coordinate from its equilibrium position. This linear coupling term allows for the mixing of different electronic states and the redistribution of electronic population among them.

The application of VC models has evolved considerably over time. Originally introduced in the 1980s, these models were primarily used to interpret vibrationally resolved bands in UV/vis absorption or ionization spectra.^21–23^ Since then, their use has expanded significantly, extending to the simulation of nonadiabatic processes,^20,24^ and the description of conical intersections in a wide range of molecular systems.^25,26^

In the early days of the VC models, the Multi-Configuration Time-Dependent Hartree (MCTDH) method^27^ was the primary tool for propagating wavepackets on these precalculated PESs.^24,28^ As a wavepacket-based approach, MCTDH can accurately describe nonadiabatic dynamics on predefined PESs and fully account for all quantum effects. However, it becomes computationally demanding for systems with many degrees of freedom due to the exponential scaling of its basis set.^29^ It is for this reason that, at the time, the use of LVC potentials enabled the first excited state dynamical simulations of systems in full dimensionality^30^—a remarkable achievement that pioneered practical applications of quantum dynamics to realistic systems.^31^ Nonetheless, MCTDH dynamics with VC potentials still formally scales exponentially, becoming too involved computationally for large systems. To alleviate this cost, the multilayer MCTDH (ML-MCTDH) method was developed.^32,33^ This approach maintains the core principles of the original method but introduces a hierarchical structure where single-particle functions are recursively decomposed into lower-dimensional functions across multiple layers. The resulting tree-like wave function representation allows flexibility in balancing numerical accuracy and computational efficiency through layer organization. While standard MCTDH uses a two-layer system (single-particle functions expanded in primitive basis functions), ML-MCTDH extends this through recursive dimensionality reduction across subsequent layers. This multilayered architecture has become particularly valuable for quantum dynamics simulations in extended molecular systems, enabling studies that would be computationally prohibitive with conventional methods.^34–38^

Trajectory surface hopping (TSH), a mixed quantum-classical (MQC) approach introduced and incrementally refined by several foundational studies^39–42^ on top of which Tully^3^ introduced the widely used fewest-switches algorithm (Figure 2a), was initially tested on one-dimensional potential energy curves.^40^ An early integration of VC models in TSH was carried out in the 90s to investigate the internal conversion in pyrazine using a two-state, three mode model.^43,44^ However, it was not until 2020 that some of us^45^ proposed the first systematic framework combining TSH with full-dimensional LVC models for polyatomic systems, yielding an practical and efficient method for simulating nonadiabatic dynamics. TSH offers a balance between accuracy and computational efficiency, making it well suitable to investigate the dynamics of large systems in full dimensionality. Taking inspiration from TSH, other trajectory-based methods have also started adopting LVC potentials to leverage their analytical simplicity and scalability.^46–49^

**Figure 2:**
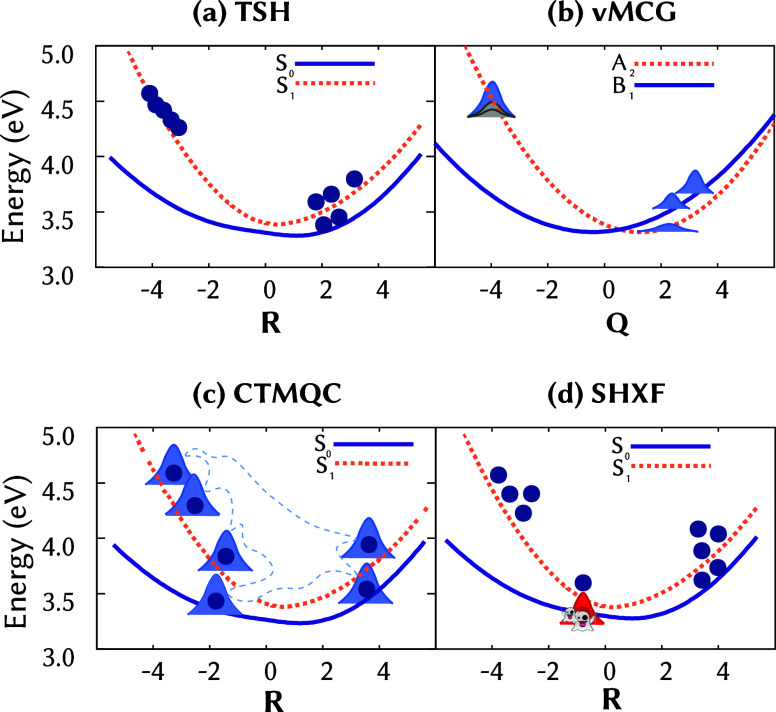
Sketch of the trajectory-based nonadiabatic dynamics methods covered in this Perspective. (a) Trajectory surface hopping (TSH), where classical nuclear trajectories (blue dots) evolve on adiabatic potential energy surfaces and probabilistically hop between them to model nonadiabatic transitions. (b) Variational multiconfiguration Gaussian (vMCG), where the nuclear wave function is represented as a superposition of Gaussian wavepackets traveling on diabatic potential energy surfaces. (c) Coupled-trajectory mixed quantum–classical (CTMQC) method, where classical nuclear trajectories evolving on adiabatic states are coupled through the quantum momentum—a term that captures electron–nuclear correlation effects. The quantum momentum is computed by placing Gaussian wavepackets on all trajectories to construct the ensemble nuclear density. (d) Exact-factorization-based surface hopping (SHXF) approach, where independent classical nuclear trajectories evolve on adiabatic states, as in standard TSH, but with additional terms that account for electron–nuclear correlation. The quantum momentum is constructed using auxiliary trajectories on nonactive states, rather than from a reconstructed nuclear density.

Variational multi-configuration Gaussian (vMCG) methods (Figure 2b) have been applied to LVC and quadratic vibronic coupling (QVC) potentials for benchmarking algorithms, implementations, and convergence thresholds.^50^ For these second-order analytical potentials, vMCG is formally exact, yielding results equivalent to MCTDH/ML-MCTDH upon basis set convergence. Beyond benchmarking, DD-vMCG (direct dynamics vMCG) extends applicability to systems requiring on-the-fly electronic structure calculations, such as systems where large amplitude motions preclude the use of VC models or systems where there is no prior knowledge of the PESs.^51^

With a similar motivation, exact factorization (EF)-based semiclassical approaches^52–58^ have recently incorporated LVC potentials. The EF framework (Figure 2c,d) offers a formally exact decomposition of the full molecular wave function into electronic and nuclear components and provides an alternative to the Born–Huang expansion for interpreting dynamical processes involving both ground and excited electronic states and their coupling through nuclear motion.^59–63^ When combined with LVC models, EF-based trajectory methods leverage analytically tractable potentials and reduced computational cost, while preserving a first-principles derivation of electron–nuclear coupling absent in traditional mixed quantum–classical approaches.^46,48,49,64^

Moreover, LVC models are also widely used as standard benchmarks for mapping-based dynamics methods.^47,65–72^ These works leverage the analytical tractability and well-defined parameter sets of LVC models to assess the accuracy of mapping approaches in predicting population transfer, branching ratios, coherence decay, and vibronic effects across a range of coupling regimes.

As a result, in recent years VC models have been increasingly adopted for running nonadiabatic dynamics simulations. While inherently limited in describing full photochemical reactivity—which may involve large-amplitude nuclear motions and bond-breaking events beyond the harmonic approximations—dynamics on VC potentials excel at capturing photophysical processes beyond the initial absorption event. LVC models have provided invaluable insights into the mechanisms governing excited-state lifetimes,^73,74^ relaxation pathways,^75–77^ charge and energy transfer events,^78,79^ exciton dynamics in molecular aggregates and condensed-phase materials,^80–83^ and, more broadly, the complex interplay between electronic and vibrational degrees of freedom in systems that otherwise would be computationally untractable with direct dynamics. Building on advances in electronic structure theory and increased computational resources, the scope of trajectory-based LVC simulations has expanded to describe relaxation dynamics and long-lived excited states over extended time scales.^84,85^ Furthermore, recent extension to solid-state systems demonstrate the applicability of LVC frameworks in condensed-phase photophysics.^86^

Within the VC framework, the spin–boson model corresponds to its most basic limit: a two-state (spin-1/2) system, interacting linearly with a bath of harmonic oscillators, under the Condon approximation where the diabatic coupling is taken to be constant.^87,88^ While the spin-boson model shares the formal structure of VC Hamiltonians, LVC models already go beyond by introducing coordinate dependence in both the diagonal and off-diagonal (coupling) elements—a minimal requirement for describing conical intersections.^20,89,90^ Due to their analytic tractability and well-characterized parameter regimes, spin-boson models are widely used as benchmarks for nonadiabatic dynamics and trajectory methods.^47,91–98^ In this perspective, however, the emphasis is put on VC Hamiltonians, which explicitly represent molecular vibronic coordinates and photochemical/photophysical processes, often involving multiple states, coordinate-dependent interstate couplings and conical intersections. A comprehensive review of spin-boson based benchmarks lies therefore beyond the scope of this work.

Overall, the role of VC models has undergone significant evolution —from their original use in interpreting absorption spectra to becoming a versatile and powerful framework for performing trajectory-based nonadiabatic dynamics simulations in large, rigid systems. Their analytical simplicity, combined with computational efficiency, makes them particularly well suited for approximating PESs and for benchmarking nonadiabatic dynamics methods on consistent, well-defined models. As a result, trajectory-based VC simulations now play a central role in enabling detailed exploration of photoactivated processes and excited-state dynamics over extended time scales. These advancements motivate the present perspective, which focuses on the use of (mostly) LVC potentials in trajectory-based nonadiabatic dynamics simulations, emphasizing their advantages as computationally efficient and analytically tractable model Hamiltonians. In doing so, it showcases how these models have enabled recent methodological developments and practical applications. The simple LVC potentials facilitate systematic benchmarking, offer clear mechanistic insights, and enable the simulation of well-chosen molecular systems over extended time scales—making them especially valuable for studying relaxation mechanisms and long-lived excited states. The use of general VC potentials not only provides an efficient route to perform explicit dynamics, but also enables the *a posteriori* evaluation of spectroscopic observables, such as pump–probe signals, directly from the trajectories—opening the door to closer comparisons between theory and experiment. Here we shall review the application of VC potentials from their renaissance with TSH—taking as a starting point the developments summarized in 2021 in ref 99—to more recent extensions, also involving vMCG and exact-factorization-based trajectory methods. Although MCTDH played a pivotal role in advancing LVC-based quantum dynamics, it lies outside the scope of this perspective, which focuses on the latest developments within the framework of trajectory-based methods. Readers interested in MCTDH dynamics on LVC potentials are referred to several comprehensive reviews available in the literature.^100–102^

The remainder of this Perspective is organized as follows. Section 2 briefly reviews the theory behind VC models. Sections 3 to 5 focus on applications where VC Hamiltonians have been used with TSH, vMCG and EF-derived methods, respectively. Section 6 discusses the limitations of VC models. Finally, Section 7 concludes with current challenges for future developments of trajectory methods based on VC potentials.

## 2 Vibronic Coupling Models

VC theory approximates the behavior of coupled PESs governing the dynamics of molecules in excited electronic states by explicitly accounting for the coupling between electronic and nuclear degrees of freedom.^22,23^ In the case of LVC, the VC model assumes a linear relationship for the coupling between electronic and nuclear coordinates. This approximation is valid for molecules that exhibit relatively small changes in molecular structure upon electronic excitation, and where the coupling between electronic and nuclear motion can be adequately described by a linear function.

The VC Hamiltonian^22,23^ expresses the PES as a Taylor expansion around a given point, normally the optimized equilibrium geometry (or Franck–Condon point) and assumes a diabatization by ansatz, i.e., the adiabatic and diabatic energies are the same at the equilibrium point. The Hamiltonian ***H*** is thus expanded as

1 $$H=H^{\left(0\right)}1+W^{\left(0\right)}+W^{\left(1\right)}+W^{\left(2\right)}+W^{\left(3\right)}+...$$

where each matrix has a total dimension corresponding to the number of electronic states involved. The zeroth order term corresponds to the kinetic energy operator

2 $$H^{\left(0\right)}={\mathrm{T̂}}_{q}=-\sum_{\alpha =1}^{n}\frac{\omega_{\alpha }}{2}\frac{\partial^{2}}{\partial Q_{\alpha }^{2}}$$

where *Q*_α_ is the normal mode displacement, ω_α_ is the ground-state normal-mode frequency, α is the normal mode index and *n* the total number of nuclear degrees of freedom.

The diagonal and off-diagonal matrix terms up to *m*th order are

3 $$\begin{array}{ll} & W_{ij}^{\left(0\right)}\left(Q\right)=E_{i}\delta_{ij}+\sum_{\alpha }^{n}\frac{1}{2}\omega_{\alpha }Q_{\alpha }^{2}\\ & W_{ii}^{\left(1\right)}\left(Q\right)=\sum_{\alpha }^{n}\kappa_{\alpha }^{\left(i\right)}Q_{\alpha }\\ & W_{ij}^{\left(1\right)}\left(Q\right)=\sum_{\alpha }^{n}\lambda_{\alpha }^{\left(i,j\right)}Q_{\alpha },i\neq j\\ & W_{ii}^{\left(2\right)}\left(Q\right)=\sum_{\alpha ,\beta }^{n}\frac{1}{2}\gamma_{\alpha ,\beta }^{\left(i\right)}Q_{\alpha }Q_{\beta }\\ & W_{ij}^{\left(2\right)}\left(Q\right)=\sum_{\alpha ,\beta }^{n}\frac{1}{2}\mu_{\alpha ,\beta }^{\left(i,j\right)}Q_{\alpha }Q_{\beta },i\neq j\\ & W_{ij}^{\left(m\right)}\left(Q_{\alpha }\right)=\frac{1}{m!}C_{\alpha }^{m,\left(i,j\right)}Q_{\alpha }^{m}\\ & W_{ij}^{\left(m+n\right)}\left(Q\right)=\frac{2}{\left(m+n\right)!}C_{\alpha ,\beta }^{m,n,\left(i,j\right)}Q_{\alpha }^{m}Q_{\beta }^{n} \end{array}$$

where *E*_*i*_ are the vertical excitation energies, κ_α_^(*i*)^ are the first-order intrastate couplings, λ_α_^(*i*,*j*)^ the first-order interstate coupling terms, γ_α,β_^(*i*)^ the second-order diagonal couplings, and μ_α,β_^(*i*,*j*)^ the second-order interstate coupling terms. An schematic representation of a VC model, including first and second-order parameters is shown in Figure 3. The vibronic coupling terms are termed *C*_α,β_^*m*,*n*,(*i*,*j*)^ in general.

**Figure 3:**
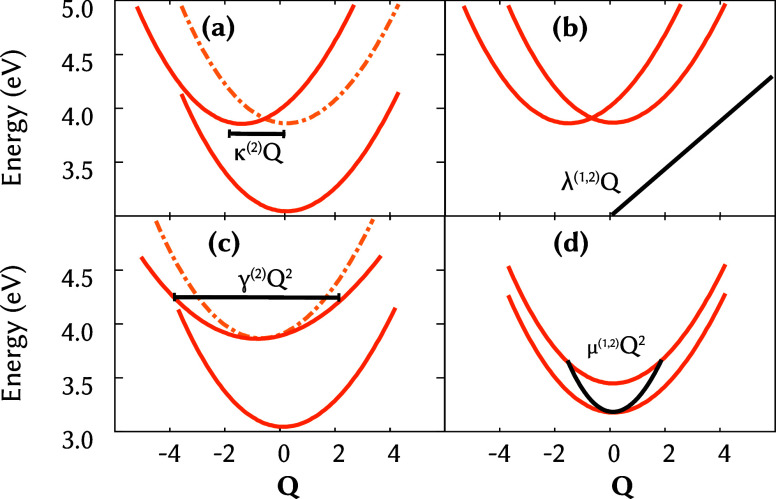
Schematic representation of the first- and second-order vibronic coupling terms of a vibronic coupling Hamiltonian model. For simplicity, the chosen normal mode displacement *Q*_α_ has been replaced by *Q*. (a) Shows the diagonal first-order term, κ_α_^(*i*)^ *Q*_*α*_, which determines how much the minimum of the excited state potential is displaced from the original vertical harmonic oscillator, $W_{ii}^{\left(0\right)}\left(Q\right)=E_{i}+\sum_{\alpha }\frac{1}{2}\omega_{\alpha }Q_{\alpha }^{2}$ , shown in dashed and softer lines. (b) The first-order off-diagonal coupling λ_α_^(*i*,*j*)^ *Q*_α_ varies linearly with the normal mode displacement *Q*_α_, connecting different electronic states *i* ≠ *j*. (c) Shows the second-order diagonal term, γ_α,β_^(*i*)^ *Q*_α_ *Q*_β_ (for α = β, γ_α,α_^(*i*)^ *Q*_α_^2^), which represents that the excited state can have a different harmonic oscillator frequency (curvature) than the ground state original, also indicated in dashed and softer lines. (d) The second-order off-diagonal coupling μ_α_^(*i*,*j*)^ *Q*_α_^2^ (for α = β) introduces a quadratic dependence on the same vibrational coordinate, representing a nonlinear interaction that modulates the coupling strength between states.

A general LVC model is built by computing the ground-state Hessian to obtain normal modes, then evaluating excited-state energies, gradients, and interstate couplings at a reference geometry. These quantities define the zeroth- and first-order terms of the Taylor expansion, namely **W** ^(0)^ (vertical excitation energies relative to the ground state) and **W** ^(1)^ (state-specific gradients and interstate couplings projected onto the normal modes). Projecting them onto the nuclear basis yields the linear vibronic coupling constants. Quadratic vibronic coupling (QVC) models additionally require **W** ^(2)^, which contains second-order terms obtained from excited-state Hessians. These Hessians can be evaluated analytically when available or approximated through finite-difference evaluation of excited-state gradients. The resulting parameters, **W** ^(0)^, **W** ^(1)^, and **W** ^(2)^, define the diabatic Hamiltonian.

However, obtaining the second-order off-diagonal terms is more challenging because it requires calculating second derivatives of the electronic wave functions with respect to nuclear coordinates (i.e., the so-called second nonadiabatic couplings).^103^ These quantities are not commonly accessible through standard quantum chemistry software packages even if it is encouraging to see that several research groups are actively developing methods in this direction.^104–108^

Instead of parametrizing at a single geometry, it is also possible to sample multiple displaced geometries along selected modes and fit the resulting excited-state properties to analytical forms, typically harmonic oscillators for potentials and linear or quadratic functions for couplings. When the potentials are harmonic and the couplings are first or second order in displacements, the model still qualifies as LVC or QVC. This multipoint fitting can improve accuracy by capturing anharmonicities and mode coupling while maintaining the formal classification of the model.

The usual approach for systems that need high-order couplings is to calculate the adiabatic energies with electronic structure packages along every single nuclear degree of freedom and fit the analytical form of a Taylor expansion with a minimum square method.^109,110^ The fitting approach, however, becomes prohibitively expensive in full dimensionality for large systems, especially if accurate quantum chemistry is used. In high-dimensional rigid systems, the one-shot approach is commonly used, where energies, gradients, and couplings are only calculated at the optimized geometries of the ground state and projected onto normal mode coordinates to calculate kappa (κ_α_^(*i*)^) and lambda (λ_α_^(*i*,*j*)^)­parameters.^45,111,112^ If analytical derivatives are not available within the electronic structure program of choice, numerical derivatives can be used, calculating points at positive and negative normal mode displacements. If that is the case, it may be possible to make use of the symmetry elements of the system and calculate only positive and negative displacements for totally symmetric modes, saving computational resources.

## 3 Trajectory Surface Hopping with Vibronic Coupling Models

In the TSH approach (Figure 2a), the nuclei are treated as classical particles that evolve on a single adiabatic PES at any given time, with stochastic hops^40^ between electronic states governed by the evolution of the time-dependent electronic wave function.^5,113,114^ Early applications of TSH and simple VC Hamiltonians include the two-state, three-mode pyrazine model, employed to investigate the dynamics of polyatomic molecules in the vicinity of a conical intersection.^43,44^ These studies demonstrated the fundamental compatibility between trajectory-based methods and LVC potentials in reduced-dimensionality systems. Building on this foundation, in ref 45 we proposed the first systematic framework that combined TSH with full-dimensional LVC models for an arbitrary number of states, within the context of the Surface Hopping including ARbitrary Couplings (SHARC) approach^115,116^ —at that time implemented in the SHARC2.1 package.^117^ It is worth noting that from a technical standpoint, performing TSH simulations on full-dimensional LVC potentials requires efficient communication between the LVC module and the dynamics driver, as otherwise massive computer time is spent in data readout. As a consequence, in our original setup,^117^ a new Python interface—PySHARC—was developed to enable in-memory communication between SHARC’s Fortran routines and the LVC module. This implementation significantly reduced overhead, thereby facilitating a more efficient execution of LVC/TSH simulations. In an effort to further simplify and enhance TSH/VC calculations, a modular implementation of SHARC has been recently developed,^118^ which allows for hybrid interfaces and thus is ideally suited to perform dynamical calculations that rely, entirely or partially on general VC potentials in a hierarchical framework. Since the first implementation of TSH/LVC in SHARC,^45^ several computational packages have subsequently incorporated VC models within TSH dynamics. Examples include the QUANTICS program^109^ using its Zagreb surface hopping interface,^119^ and other TSH packages that support simulations on analytical model surfaces, such as Newton-X,^120^ Libra,^121^ JADE,^122^ PyUNIxMD^123^ or CTQMC.^49,124^

A key strength of the efficiency provided by VC frameworks is that they facilitate methodological insights into TSH simulations by critically assessing the accuracy and robustness of different TSH protocols. An early example was provided by Plasser et al.^125^ who systematically evaluated the influence of two decoherence correction schemes and various strategies for momentum rescaling and treating frustrating hops during the dynamics of [Re­(im)­(CO_3_(phen)]^+^ (im = imidazole, phen = phenanthroline). There, a total of 13 different TSH protocols were prepared and compared against an MCTDH reference, using a reduced LVC model of 15 degrees of freedom with two singlet and four triplet states. The 200 trajectories propagated over 500 fs would correspond to more than 10 million formal electronic structure computations—practically unfeasible with direct dynamics, but easily affordable on LVC preparameterized potentials. Their findings highlighted the importance of a careful protocol design, with momentum rescaling along a single mode and momentum reflection after frustrated hops emerging as particularly effective.^125^ Further methodological challenges, such as the treatment of decoherence^126^ or how to leverage LVC/TSH dynamics to guide dimensionality reduction,^127^ were reviewed up to 2021 in ref 99.

Similarly, LVC potentials can also be used to assess the influence of different levels of electronic structure theory on the dynamics. For example, ref 128 investigated the impact of various parametrizations of a range-separated functional on the dynamics of the [Fe­(cpmp)]^2+^ complex (cpmp = 6,2′-′carboxypyridyl-2,2′-methylamine-pyridylpyridin), revealing dramatic differences despite the absorption spectra being similar across the investigated DFT methods. In this context, a protocol to generate LVC potentials from the Green’s Function - Bethe-Salpeter equation approach has also recently been introduced and applied to the same Fe­(II) compound,^129^ in combination with ML-MCTDH.

In a recent study, the reliable parametrization of LVC models—with emphasis on systems with many degrees of freedom and near-degenerate states—was recently examined by some of us,^130^ underscoring the importance of careful numerical differentiation, consistent phase correction and high-precision electronic structure calculations. To conclude methodological aspects, we mention a comprehensive review from Faraji and co-workers,^131^ who surveyed recent advances in excited-state dynamics, highlighting the importance of hybrid quantum–classical approaches, including TSH on VC models, for simulating photoinduced processes in complex environments.

On the application side, the number of molecular systems investigated with TSH/LVC dynamics is rapidly increasing. As evident from the examples mentioned above, LVC models are particularly effective for simulating nonadiabatic dynamics in transition metal complexes, where dense manifolds of spin- and electronically excited states often challenge ab initio methods. Beyond early applications discussed in ref 99, full-dimensional TSH/LVC simulations on Fe­(II) complexes^73,132–134^ have provided insights into the ultrafast deactivation mechanisms of photosensitizers, capturing coherent vibrational dynamics in close agreement with femtosecond (X-ray) experimental data. These studies were primarily based on DFT energies, gradients and couplings, except that in the last example^134^ CASPT2 spin–orbit couplings were incorporated into the otherwise DFT PESs to describe deactivation from the triplet to quintet states in ${\left[{Fe\left(terpy\right)}_{2}\right]}^{2+}$ (terpy = 2,2′:6′,2″-terpyridine). DFT-based TSH/LVC simulations have also been employed in a rhenium-sensitized azurin protein providing a detailed description of ultrafast intersystem crossing and charge separation in bioinorganic systems and supporting experimental evidence for subpicosecond formation of charge-separated states.^78^ In the [Ru­(^*S*^^–^^*S*^bpy)­(bpy)_2_ ]^2+^ complex, DFT-based TSH/LVC simulations in combination with transient absorption spectroscopy revealed that, regardless of the initial excitation wavelength, rapid intersystem crossing leads to charge localization on the sulfur-decorated ligand and the formation of a single long-lived species within 80 fs.^79^

A key advantage of parametrizing VC models is that they allow the use of highly accurate, albeit computationally demanding multiconfigurational methods, to compute all the required parameters (energies, forces and couplings). Multiconfigurational electronic structure methods, such as CASSCF and CASPT2, are essential for reliably describing open-shell transition metal complexes, among other challenges, where a dense manifold of low-lying electronic states must be taken into account; however, they would be computationally prohibitive in direct dynamics simulations. In this context, TSH/LVC dynamics has enabled detailed simulations of the spin photodynamics of open-shell Cr­(III) and V­(III) systems, where CASSCF-based LVC potentials revealed state-specific intersystem crossing pathways and vibrational modes driving the relaxation.^76,84^ A follow-up screening of V­(III) emitters systematically investigated four additional complexes selected among 18 derivatives with the aim to increase the ligand-field splitting and enhance phosphorescence emission in the near-infrared region.^135^ Similarly, CASSCF-based TSH/LVC dynamics including implicit solvation applied to Ru-based Creutz–Taube ion analogue revealed a long-lived mixed-valent excited state and persistent metal–bridge vibrational coherence beyond 1 ps.^136^

TSH/LVC methodologies can also be applied to nonmetal systems, provided that the system is sufficiently rigid to justify the harmonic approximation of the underlying potentials. While direct dynamics might seem preferable for organic photophysics, LVC parametrizations become advantangeous in systems involving many quasi-degenerate excited states and pronounced spin–orbit couplings. In acetone, TSH/LVC simulations were used to resolve ultrafast population exchange among nearly degenerate Rydberg states,^75^ as observed with time-resolved photoelectron spectroscopy. TSH/LVC dynamics of brominated eumelanin monomers (DMICE-Br2 and DMICE-Br3) revealed subpicosecond ISC enhanced by heavy atom effects, consistent with transient absorption data.^77^ Xie et al.^86^ presented a TSH methodology for simulating photoinduced carrier relaxation in periodic solids, based on a LVC model parametrized from Kohn–Sham orbital energies and density functional perturbation theory with Wannier interpolation applied to obtain electronic and vibrational couplings across the Brillouin zone. This approach bridges nonadiabatic dynamics with solid-state electronic structure, enabling simulations of ultrafast processes in crystalline materials.

The use of VC potentials also facilitates the incorporation of explicit laser fields in the dynamics. While this is straightforward in grid-based wavepacket methods—where the dipole approximation allows the time-dependent interaction with an external electromagnetic field to be included directly in the Hamiltonian—such implementations remain rare in TSH approaches.^115,137–140^ In particular, previous studies^141,142^ have shown that standard TSH suffers from fundamental limitations when combined with extended or intense laser pulses, such as energy conservation, the inability to correctly describe light–matter coherence, and unreliable treatment of decoherence—especially problematic for long pulse durations or resonant excitation. The use of VC potentials offers practical advantages that partially alleviate the challenges involved in laser-driven TSH simulations. The diabatic representation of VC models lead to smoother nuclear dynamics and help to avoid the gauge dependence and numerical instability. The harmonic nuclear PES in VC models can partially offset the known decoherence deficiencies in standard TSH, especially when strong laser fields perturb the wavepacket. Among other difficulties inherent to the nature of TSH, simulating laser-driven dynamics with TSH is particularly demanding because, under realistic experimental conditions, only a small fraction of the molecular ensemble—often just a few percent—is actually excited by the laser pulse. This implies that a large number of trajectories must be propagated to ensure sufficient statistical sampling of the excited-state dynamics. To avoid this burden, TSH dynamics typically assumes that the system is promoted to the relevant excited states instantaneously, with 100% population transfer at time zero —effectively modeling a delta-pulse excitation. The computational efficiency of VC models enables propagation of large trajectory ensembles, which is essential for sampling the small excited-state population typically involved in an explicit laser excitation. These features make VC models a useful platform for testing and benchmarking TSH under explicit field conditions. While limitations persist for long pulses or strong-field regimes, use of VC potentials allows exploration of light–matter interaction effects in trajectory-based dynamics in a controlled and computationally affordable manner. VC models offer a clear advantage by allowing the propagation of thousands of trajectories at a fraction of the cost of fully on-the-fly calculations. In 2021, some of us^143^ evaluated the performance of TSH/LVC under laser excitation by comparing it to MCTDH dynamics for SO_2_ and 2-thiocytosine, identifying the treatment of electronic coherence as a key factor influencing agreement with the quantum benchmarks. Later, we demonstrated with TSH that explicit, tailored laser pulses can steer the excited-state dynamics of the [Ru­(^*S*^^–^^*S*^bpy)­(bpy)_2_ ]^2+^ complex, effectively suppressing natural S–S bond dissociation by exploiting laser-induced transient population dynamics along triplet-state pathways.^144^

By construction, TSH/LVC dynamics is intended for use either in the gas phase or with an implicit solution—the latter simply by replacing gas phase energies *E*_*i*_ by those computed with an implicit model. Explicit solvent models via hybrid quantum mechanical/molecular mechanics (QM/MM) schemes are not directly compatible with LVC models because the interaction between the QM region and the MM environment usually described via electrostatic embedding requires the point charges from MM to act on the QM density; however, there is no electron density in LVC and thus no standard, rigorous way to incorporate MM degrees of freedom into the VC Hamiltonian. A promising development introduced by Polonius et al.^145^ is an hybrid LVC/MM framework, which extends VC models to simulate photoinduced nonadiabatic dynamics of solvated systems. This approach incorporates electrostatic embedding via distributed multipole expansions, up to quadrupoles, to model interactions between the solute’s diabatic states and the MM environment. Benchmark LVC/MM simulations on thioformaldehyde in explicit water reproduced solvation structures and energetics with 1–2 kcal/mol accuracy versus QM/MM. Follow-up LVC/MM studies have been applied to thiocarbonyls in water^146^ and to solvated [Fe­(CN)_4_(bipy)]^2 –^ (bipy = 2,2′-bipyridine), including all linear and some selected quadratic coupling terms.^147^ These studies successfully modeled explicit hydrogen-bond rearrangements and anisotropic solvent–solute dynamics using large-scale trajectory ensembles.

To conclude this section, we mention studies that combine TSH/LVC with (ML-)­MCTDH, as a powerful hybrid strategy that capitalizes on the complementary strengths of both methods: TSH/LVC enables fast, full-dimensional exploration of nonadiabatic dynamics and helps identify the key vibrational modes and couplings that govern relaxation, while (ML-)MCTDH provides a highly accurate quantum description of the selected degrees of freedom. One example is the iterative dimension reduction scheme that integrates TSH/LVC and MCTDH within a feedback loop, where TSH guides the identification of the most relevant nuclear modes until the vibronic Hamiltonian becomes amenable to a quantum dynamics treatment.^127^ This strategy has been adopted in subsequent studies,^148,149^ where SHARC-based TSH simulations were used to select optimal normal modes for subsequent MCTDH calculations on transition metal complexes.

Comparisons between TSH/LVC and alternative nonadiabatic methods underscore that each approach involves trade-offs between different strengths, such as statistical sampling, decoherence treatment, or access to quantum coherence. The optimal choice ultimately depends on the specific characteristics of the system under study. Precomputed LVC potentials have also been employed in ML-MCTDH full quantum dynamics frameworks to simulate polycyclic aromatic hydrocarbons,^36^ highlighting the compatibility of LVC-based models with multiple dynamic schemes. When carefully parametrized, TSH/LVC can reproduce key observables from fully on-the-fly dynamics with high fidelity, particularly population dynamics, state-to-state branching ratios, and qualitative nuclear relaxation trends. Differences can still arise in observables that are sensitive to electronic coherence or anharmonic nuclear motion. For instance, studies on Ru- and Rh-based photosensitizers,^74,130^ have shown that TSH/LVC simulations captured key dynamical features in close agreement with direct dynamics. Nonetheless, the results are not strictly identical, and system-specific considerations remain critical when selecting the appropriate simulation method.

## 4 Variational Gaussians on Vibronic Coupling Potentials

Pioneered by Heller in the 1970s,^4^ the use of a wave function ansatz based on a linear combination of frozen Gaussians to simulate the time evolution of a nuclear wavepacket (see Figure 2b) was later independently developed by Truhlar,^150^ Buch,^151^ and Metiu.^152^

Spawning-based frozen-Gaussian wavepacket methods were used in the late 1990s by Martínez, Ben Nun, and Levine.^153–156^ Full Multiple Spawning (FMS)^153,154^ introduced the idea of representing the nuclear wave function as a linear combination of frozen Gaussian trajectory basis functions, whose centers evolve along classical trajectories, with new Gaussians spawned adaptively in regions of strong nonadiabatic coupling. The Ab Initio Multiple Spawning (AIMS) method^155,156^ extended FMS to on-the-fly electronic structure calculations while retaining the same spawning framework. Subsequently, Worth and Burghardt introduced the vMCG method,^157^ which originated from the MCTDH^158^ family and explicitly evolves Gaussians variationally rather than classically. In parallel, Shalashilin and co-workers developed the multiconfigurational Ehrenfest (MCE)^92^ and coupled-coherent-states approaches,^159,160^ which propagate a superposition of trajectory-guided Gaussian coherent states via a time-dependent variational principle; on-the-fly ab initio variants (AI-MCE) and two-layer coupled-coherent-states formulations have been reported and applied to prototypical LVC benchmarks such as pyrazine.^161–163^ Building on concepts from MCE and AIMS, the ab initio multiple cloning method was introduced to capture wavepacket branching by propagating Ehrenfest-guided coherent states and cloning them adaptively in regions of strong interstate mixing, while retaining on-the-fly electronic structure.^92,164–166^

The vMCG method consists of a frozen Gaussian wave function ansatz

4 $$\Psi \left(Q,t\right)=\sum_{i=1}^{N}A_{i}\left(t\right)G_{i}\left(Q,t\right)$$

where *Q* is the coordinate of the Gaussian center, normally expressed as normal mode coordinates, and the gaussians *G*_*i*_(*Q*, *t*) are the product of one-dimensional basis functions, i.e. (*g*_*j*_^(1)^(*Q*_1_,*t*)...*g*_*j*_^(*f*)^(*Q*_*f*_,*t*)), each one of them taking the form

5 $$g_{i}^{\left(\alpha \right)}\left(Q_{\alpha },t\right)=\exp\left(-\beta_{i}^{\left(\alpha \right)}Q_{\alpha }^{2}+\xi_{i}^{\left(\alpha \right)}\left(t\right)Q_{\alpha }+\eta_{i}^{\left(\alpha \right)}\left(t\right)\right)$$

where β represents the fixed widths of the functions (as assumed in the frozen Gaussian approximation), η are scalar parameters chosen to ensure the functions remain normalized and free of phase, and ξ_*i*_ are the linear parameters that encapsulate the time dependence of the functions.

This wave function ansatz is then introduced in the Dirac-Frenkel variational principle to solve the time-dependent Schrödinger equation, leading to the following equations of motion for the expansion coefficients

6 $$i{Ȧ}_{i}=\sum_{jk}{\left\langle G_{i}|G_{j}\right\rangle }_{ij}^{-1}\left(\left\langle G_{i}\left|H\right|G_{j}\right\rangle -i\left\langle G_{i}|{Ġ}_{j}\right\rangle \right)A_{k}$$

where the term ⟨*G*_*i*_|*G*_*j*_⟩ are the Gaussian function overlaps *S*_*ij*_, the term ⟨*G*_*i*_|*H*|*G*_*j*_⟩ are the Hamiltonian matrix elements *H*_*ij*_ and the term $\left\langle G_{i}|{Ġ}_{j}\right\rangle$ is the overlap time derivative.

The Gaussian parameters evolve as follows

7 $$i{\mathrm{\xi ̇}}_{\alpha i}=-2\beta \frac{\partial V}{\partial Q_{\alpha }}+i\frac{p_{\alpha i}}{m_{\alpha }}+\sum_{\mu j}\rho_{ij}\left(S_{ij}^{\alpha \mu }-S_{ik}^{\left(\alpha 0\right)}S_{kl}^{-1}S_{lj}^{\left(0\mu \right)}\right)\sum_{\mu j}\left(i\rho_{ij}H_{ij}^{0\mu }-\rho_{im}S_{ik}^{\left(\alpha 0\right)}S_{kl}^{-1}S_{lj}^{\left(0\mu \right)}H_{jm}\right)$$

One could see the centers of the Gaussian wave packets (GWP) functions as following “trajectories” with a classical part $-2\beta \frac{\partial V}{\partial Q_{\alpha }}+i\frac{p_{\alpha i}}{m_{\alpha }}$ and a quantum coupling (the third term in the sum, also simplified as *C* ^–1^ *Y*_*R*_) which couples the GWP variationally, driving them to the optimal place in the configurational space. If the *H*_*ij*_^(αβ)^ matrix elements are evaluated exactly the result converges to the numerical solution of the time-dependent Schrödinger equation with conservation of energy and norm by construction. However, when used on-the-fly, with only local information on the GWP known at each time step, the integrals over the potential operator are approximated to second order, which is known as the local harmonic approximation.^157^ In this approximation, the PESs are expanded to second order around the coordinate at the center of the GWP

8 $$V\left(Q\right)\approx V\left(Q_{j}\right)+\sum_{\kappa }{\frac{\partial V}{\partial q_{\alpha }}|}_{Q_{j}}\left(Q_{\alpha }-Q_{\alpha j}\right)+\frac{1}{2}\sum_{\alpha \alpha^{\prime }}{\frac{\partial^{2}V}{\partial Q_{\alpha }Q_{\alpha^{\prime }}}|}_{Q_{j}}\left(Q_{\alpha }-Q_{\alpha j}\right)\left(Q_{\alpha^{\prime }}-Q_{\alpha^{\prime }j}\right)$$

where *Q*_*j*_ is the center coordinate of the Gaussian *G*_*j*_. This allows the integral to be calculated analytically. This method provides exact integrals if the potential is of lower or equal order than the expansion, i.e., for LVC models or QVC models.

DD-vMCG is typically used in an on-the-fly manner, calling an external electronic structure package and allowing for more flexible forms of the PES (recall Figure 1a). In this approach, a database of electronic structure points is built as the simulation progresses. Notably, after calculating just the first point, we already have enough information to construct a (quadratic) vibronic coupling Hamiltonian. This is very useful for testing, as it allows us to directly compare DD-vMCG with TSH and MCTDH based methods on the same PESs. The vMCG method is propagated on diabatic surfaces (same as MCTDH based methods) and the surfaces are usually constructed with the propagation diabatization algorithm.^167^

The vMCG method was first presented in 2003, applied to a multistate system of the butatriene cation, where the PESs were approximated using a VC model.^168^ In that work, the authors compared nonadiabatic dynamics results with previous TSH calculations,^169^ finding similar outcomes but with vMCG allowing a fully quantum treatment of the nuclei.

A summary of the vMCG’s capabilities was provided in ref 157, demonstrating its flexibility to describe dynamics in pyrazine (with a VC Hamiltonian), salicylaldimine proton transfer (on-the-fly), and butatriene (with an VC Hamiltonian). The pyrazine VC model was used again in 2019 by Penfold et al.,^170^ to evaluate the performance of explicit laser pulses in the dynamics compared to MCTDH. The VC Hamiltonian was also used to study allene radical cation dynamics with vMCG,^171^ and this was revisited in 2021 by Christopolou et al.,^172^ who demonstrated improved performance using a new interpolation scheme for on-the-fly calculations.

It is important to note that vMCG is mainly used with VC potentials to benchmark new features, implementations, algorithms, or threshold parameters. As mentioned before, for analytical potentials up to second order (LVC and QVC), the local harmonic approximation used to evaluate integrals is formally exact and should yield the same results as MCTDH or ML-MCTDH when the basis set is converged. Using ML-MCTDH helps reducing the exponential scaling of MCTDH, and the computational cost of variational Gaussians becomes similar or only slightly higher. In order to handle more flexible systems, vMCG was extended to incorporate on-the-fly electronic structure calculations through the interaction of quantum chemistry software (Figure 1a). In this context, DD-vMCG has been used to study the photodissociation of methanol^51^ and *cis*–*trans* photoisomerizations,^50,173^ which cannot be described by a VC model due to large amplitude motions.

In recent years, the nonadiabatic dynamics community has begun joint efforts to establish benchmark systems that researchers can use to test their codes and set standards for the field.^174^ One of the first steps was to remove the electronic structure variable and test methods on the same set of PESs.^175^ In 2021, the groups led by Worth and Doslic implemented TSH (the Zagreb package^119^) in the QUANTICS package,^109^ performing benchmark calculations on the butatriene cation (with a VC Hamiltonian) and the cyclohexadiene molecule (on-the-fly), showing stable and comparable results.^119^

Similarly, the Tully models are 1D systems proposed by Tully^3^ for testing TSH. In 2020, Ibele and Curchod^176^ proposed real molecules, namely ethylene, fulvene, and DMABN, as prototypes whose photophysics resemble those models. In 2024, Gómez, Spinlove, and Worth applied the same level of theory to these systems using the DD-vMCG method, also running dynamics on (Q)­VC models generated from the first time-step of the dynamics.^50^ For DMABN and fulvene, the VC results reproduced the main photodeactivation pathways at a much lower computational cost. A few years ago, Worth and Gómez showed that for DMABN in the gas phase and in three solvents, the VC model Hamiltonian produced qualitatively correct dynamical trends.^177^ Also in 2024, a new approach was implemented to use vMCG with parametrized VC potentials from force fields,^178^ although a practical application including explicit solvent molecules is still in progress.

## 5 Exact Factorization and Linear Vibronic Coupling Models

The EF approach^59–61^ has recently offered a new perspective on nonadiabatic dynamics. By providing a formally exact splitting of the electronic and nuclear degrees of freedom, EF captures electron–nuclear correlation through coupled equations of motion for the electronic and nuclear wave functions. This framework offers a rigorous starting point for constructing approximations and has motivated the development of several MQC methods aimed at incorporating nonadiabatic effects and decoherence from first principles. Based on systematic approximations for classical nuclei, different EF-based MQC approaches have been proposed. These include the coupled-trajectory mixed quantum–classical (CT-MQC) methods^52–56^ (Figure 2c), which incorporates quantum features of the nuclear distribution through coupled classical trajectories, the surface hopping based on exact factorization (SHXF) approach (Figure 2d),^57,58,123^ an independent-trajectory method with further approximations for computational efficiency and numerical stability, and the quantum-trajectory surface-hopping approach with the electronic equation derived from the exact-factorization approach QTSH-XF,^179^ a method that eliminates the ad hoc aspects from TSH: the velocity rescaling, the treatment of frustrated hops and the decoherence correction. Compared to traditional Ehrenfest and TSH methods, EF-based MQC schemes include additional terms in the electronic and nuclear equations that capture electron–nuclear correlation more accurately and describe decoherence^52–54,57,123,180,181^ and electronic coherences^182^ from first principles.

LVC models have played a key role in benchmarking EF-based methods, offering a controlled and computationally efficient setting to assess the impact of these additional terms, particularly in capturing the correct coupled electron–nuclear dynamics through conical intersections. In a first study by Vindel-Zandbergen, Matsika and Maitra,^46^ a LVC model for the uracil cation featuring a three-state conical intersection was employed,^183,184^ showing that TSH with the EF electronic equation closely reproduced MCTDH results, whereas standard decoherence-corrected surface hopping failed. The LVC model allowed for a clear and systematic comparison between MQC and MCTDH dynamics, allowing for the explicit computation of the nonadiabatic dynamics within a reduced yet representative model space. The LVC model was constructed from EOM-IP-CCSD electronic structure calculations and the computed photoelectron spectrum was compared against experimental data.^185^ The model included both two- and three-state conical intersections obtained at the EOM-IP-CCSD level, which are essential for capturing the key photophysical relaxation pathways of the uracil cation. The LVC model allowed the investigation the dynamics through the three-state intersection-an especially challenging scenario for MQC methods—while retaining the accuracy of the EOM-IP-CCSD description for PESs. The SHXF was shown to outperform traditional MQC schemes when a given trajectory is associated with more than two Born–Oppenheimer states simultaneously during the dynamics (multistate dynamics) because of quantum-momentum-driven transitions that are missing in the standard approaches. In refs 49,186 , the same LVC model was used to further investigate EF-based approximations, including the use of coupled versus auxiliary trajectories in evaluating the electron–nuclear correlation terms, the approximation of using these terms within SH and Ehrenfest frameworks, and the relevance of the exact conditions of zero population transfer away from nonadiabatic coupling regions and energy conservation. Additionally, quantum-trajectory surface-hopping approach with the electronic equation derived from the exact-factorization approach (QTSH-XF), proposed by Dupuy, Rikus and Maitra,^179^ was also examined and compared to SHXF using different momentum rescalings. Another example involves a set of two-dimensional two-state LVC models representing molecular systems with a conical intersection: bis­(methylene) adamantyl radical cation, the butatriene cation, and pyrazine. These systems were initially studied using trajectory-based methods, including ab initio multiple spawning and TSH,^64^ to analyze the dynamics through conical intersections. More recently, they have been employed to test a modified version of the CTMQC method, referred to as CTMQC-E, in which specific terms were redefined to ensure energy conservation.^49^ Originally, these LVC models were introduced to explore the role of the geometric phase in internal conversion processes.^187^ The use of LVC models has greatly facilitated the testing of new derivations of EF-based trajectory methods and their comparison against traditional trajectory-based strategies (such as surface hopping and Ehrenfest), including various rescaling procedures and decoherence corrections—a task that would have been unimaginable with direct dynamics due to the prohibitive computational cost. Although LVC models have not been as extensively used in the context of EF-based methods compared to TSH—owing to the relatively recent development and computational implementation—they offer a valuable platform for testing and benchmarking new implementations.^48^

## 6 Limitations of Vibronic Coupling Models

Although VC models—particularly in their linear (LVC) form—have proven invaluable for simulating nonadiabatic excited-state dynamics, they remain subject to inherent approximations that limit their range of applicability. Their main limitation lies in the assumption of a linear relationship between the electronic and nuclear degrees of freedom. This approximation breaks down in systems with large amplitude motions or strong vibronic coupling, where nonlinear effects become significant. In such cases, the LVC model may fail to accurately capture the nonadiabatic effects and the full-dimensional excited-state dynamics can be compromised. If the normal modes corresponding to the large amplitude motion are not critical for the relaxation, e.g. methyl rotations, they can be excluded and the resulting LVC model might still be effective despite being of lower dimensionality.^79^ Recently, Penfold and Eng^112^ developed the Global Anharmonicity Parameter method, which provides a quantitative measure of the breakdown of the LVC potentials by considering anharmonic effects, calculating the difference between the true excited state minimum geometry and the geometry obtained using the LVC model, providing insights into the validity of the LVC model and the level of anharmonicity in the system. Approaches beyond the linear approximation include employing quadratic (QVC) or higher-order terms or selectively replacing the poorly described normal modes with internal or curvilinear coordinates, while keeping the remaining LVC framework. Naturally, as the VC models increase in complexity, the fitting becomes more tedious and computationally demanding, necessitating more sophisticated parametrization strategies and efficient algorithms to maintain their practical applicability. Strategies to handle increased complexity in VC models could include machine learning approaches, modular fragment-based fitting, and automated optimization algorithms.

As an alternative development, machine learning potentials can be used not only to augment LVC models but to replace them altogether.^188^ Trained on high-level quantum chemical data, machine learning models are capable of capturing anharmonicities and nonlinear couplings with high accuracy across a broad configurational space, while retaining computational efficiency. In hybrid schemes, machine learning potentials may also be employed to correct residual errors in LVC parametrizations, thereby extending the applicability of VC-based dynamics to more complex or flexible systems.^189,190^

As with any other dynamical method,^7^ the reliability of the results is ultimately dictated by the level of theory in the electronic structure determines employed. LVC models rely on parametrizations obtained from quantum-chemical calculations, which can still be computationally demanding for large or complex systems and often exhibit errors exceeding chemical accuracy (1 kcal/mol). Because parametrizing LVC models is far cheaper than performing on-the-fly, direct dynamics, this low cost should encourage the use of high-quality electronic-structure data rather than cheaper methods that could compromise the accuracy of the resulting potentials and dynamics. Besides the intrinsic error of the potentials, one should also realize that LVC is limited due to the truncation to only several low-lying electronic states. Adding more adiabatic states allows for the construction or more accurate diabatic VC potentials, which should better capture state mixings arising from nonadiabatic or spin–orbit couplings.

## 7 Conclusions

In this Perspective, we have highlighted the significant role of vibronic coupling (VC) potentials—especially the linear vibronic coupling (LVC) model—in advancing trajectory-based excited-state dynamics simulations. By providing a computationally efficient yet physically insightful framework, VC models bridge the gap between accurate electronic structure theory and the complex nuclear dynamics that govern photoinduced processes. The superior efficiency of VC potentials for trajectory-based dynamics against direct or on-the-fly dynamics enables significantly longer propagation times, larger ensemble of trajectories and the use of more accurate electronic structure methods. These advantages make it feasible leveraging trajectory methods in scenarios that would be computationally prohibitive using on-the-fly dynamics. Examples include carrying out dynamical studies in systems that require expensive multiconfigurational methods, the inclusion of explicit laser fields, benchmarking the influence of different electronic structure methods on the dynamics, or assessing the impact of various dynamical parameters on the resulting dynamics in a systematic and controlled manner.

We discussed the integration of VC models with three trajectory-based dynamical methods: surface hopping, variational multiconfigurational Gaussian, and exact-factorization-based approaches. Since its initial implementation for dynamics with surface hopping,^45^ and our first review,^99^ the past years have witnessed a significant expansion in the application of VC models for nonadiabatic dynamics. Table 1 collects the most recent systems studied in this fashion. VC potentials are clearly gaining in importance, but fundamental challenges remain that limit their broad application. Despite their strengths, VC models have inherent limitations, particularly in capturing large-amplitude motions and strong anharmonicities. This motivates ongoing developments incorporating quadratic terms, mixed coordinates, and machine learning corrections. To fully realize the potential of VC potentials, alongside methodological improvements, efforts must address challenges spanning from accurate parametrization to efficient integration with advanced dynamical frameworks. Future developments toward the explicit simulation of time-resolved spectroscopies are also possible,^191–194^ opening the door to predictive comparisons with ultrafast experiments.

**Table 1:** Molecular Systems Discussed in the Manuscript, Grouped by Trajectory-Based Nonadiabatic Dynamics Method, Type of Vibronic Coupling Hamiltonian (“No” Refers to On-the-Fly Dynamics) and Reference

molecular system	category	NAMD method	VC model	ref
acetone	organic	TSH	LVC	^75^
DMICE-Br2, DMICE-Br3	organic	TSH	LVC	^77^
periodic solids (Hua Xie et al.)	solid-state	TSH	LVC	^86^
SO_2_, 2-thiocytosine	organic	TSH	LVC	^143^
[Ru(^S–S^bpy)(bpy)_2_]^2+^	metal complex	TSH	LVC	^144^
Fe(II) photosensitizers	metal complex	TSH	LVC	^132–134^
thioformaldehyde in water	solvated organic	TSH	LVC/MM	^145^
thiocarbonyls, iron complex	solvated/metallic	TSH	LVC/MM	^146,147^
polycyclic aromatic hydrocarbons	organic	ML-MCTDH	LVC	^36^
Ru- and Rh-based photosensitizers	metal complex	TSH	LVC	^74,130^
butatriene cation	organic	vMCG	VC	^168^
pyrazine	organic	vMCG/TSH/AIMS/CTMQC(-E)	VC	^49,157,195^
BMA	organic	AIMS/TSH/CTMQC(-E)	LVC	^49,195^
salicylaldimine	organic	DD-vMCG	no	^157^
allene radical cation	organic	vMCG	VC	^171,172^
butatriene cation	organic	TSH/AIMS/CTMQC(-E)	VC	^49,119,195^
methanol	organic	DD-vMCG	no/LVC	^51^
PSB3	organic	DD-vMCG	no	^173^
ethylene, fulvene, DMABN	organic	TSH/(DD)-vMCG	no/LVC/QVC	^50^
DMABN (gas/solvent)	organic	(DD)-vMCG	no/LVC	^177^
fulvene, DMABN	organic	CTMQC, (C)CTTSH	LVC	^48^
uracil	organic	vMCG	LVC/MM	^178^
uracil cation	organic	MCTDH/CTMQC(-E)/SHXF/QTSH-XF	LVC	^46,49,179^
